# Identifying and assessing the potential hydrological function of past artificial forest drainage

**DOI:** 10.1007/s13280-017-0984-9

**Published:** 2017-11-02

**Authors:** Eliza Maher Hasselquist, William Lidberg, Ryan A. Sponseller, Anneli Ågren, Hjalmar Laudon

**Affiliations:** 1 Department of Forest Ecology and Management, Swedish University of Agricultural Sciences (SLU), Skogsmarksgränd, 901 83 Umeå, Sweden; 2 Department of Ecology and Environmental Science, Umeå University, 901 87 Umeå, Sweden

**Keywords:** DEM, Flow accumulation model, Hydrology, LiDAR, Peatland, Terrain-based prediction

## Abstract

Drainage of forested wetlands for increased timber production has profoundly altered the hydrology and water quality of their downstream waterways. Some ditches need network maintenance (DNM), but potential positive effects on tree productivity must be balanced against environmental impacts. Currently, no clear guidelines exist for DNM that strike this balance. Our study helps begin to prioritise DNM by: (1) quantifying ditches by soil type in the 68 km^2^ Krycklan Catchment Study in northern Sweden and (2) using upslope catchment area algorithms on new high-resolution digital elevation models to determine their likelihood to drain water. Ditches nearly doubled the size of the stream network (178–327 km) and 17% of ditches occurred on well-draining sedimentary soils, presumably making DNM unwarranted. Modelling results suggest that 25–50% of ditches may never support flow. With new laser scanning technology, simple mapping and modelling methods can locate ditches and model their function, facilitating efforts to balance DNM with environmental impacts.

## Introduction

Artificial drainage of forested wetlands and peatlands that increase forest production has profoundly altered the hydrology of North-European landscapes during the twentieth century (Rydin and Jeglum 2009). Near-surface water saturation of soils reduces gas exchange with the atmosphere and thus soil oxygen availability, which in turn impairs the function of plant roots of many species (Sikström and Hökkä 2016). Ditching lowers the ground water level (GWL), increases the depth of the unsaturated zone, and makes conditions more favourable for tree roots. Thus, ditching can increase tree growth if other factors are not limiting, e.g. nutrients (Sikström and Hökkä 2016). Drainage for forestry has been most intense in Europe, affecting at least 20% of peatland areas (Rydin and Jeglum 2009). The greatest drained areas used for forestry have been in Russia and the Baltic States where over 13.5 million hectares of wetlands have been ditched (Paavilainen and Päivänen 1995); in Canada, similar approaches are being considered (Lavoie et al. 2005).

In the Nordic countries, peatlands have been drained for forestry since the late 1800s or early 1900s (Lundberg 1914). In Sweden, based on the notion that any mire could be turned into a productive forest—and through support from a public works relief programme—state subsidies were granted to private landowners to drain peatlands and wet forests with a peak in ditching during the 1930s (Päivänen and Hånell 2012). During World War II, government funding was reduced, leading to a general decline in the creation of new ditches. During the 1980s, environmental problems associated with ditching gained attention and consultation with the Forestry Board in Sweden was required to create new ditches. Eventually, a permit was required to do this. Now, construction of new forest ditches has virtually ceased due to requirements for Forest Stewardship Council certification (FSC 2010). In Finland, state subsidies also began in the 1930s, stopped during the war, and then peaked between 1950 and 1970 (Päivänen and Hånell 2012). Currently no new peatland areas are being drained in Finland, but new ‘complementary ditches’ are often dug next to older ditches to maintain drainage (Sikström and Hökkä 2016).

The point of this historical perspective is that most ditches in Sweden were dug before modern mapping techniques and predate the living memory of residents. Finland, on the other hand, dug most ditches 30–40 years later than Sweden and have been actively managing them since (Päivänen and Hånell 2012). Thus, Sweden is left in a situation where many ditches were dug because people were paid to dig them, and these are not always easy to locate on the landscape. Thus, forest managers cannot evaluate if they were planned well in the first place and if or how they should be managed.

Ditches have fundamentally changed the rate, amount, and quality of water as it moves through a catchment, both above and below ground. When wetlands are ditched or shallow drainages are channelised, the flow of water can become several orders of magnitude faster than matrix and macropore flow through soils (Rawls et al. 1993), the dominant flow paths for water in saturated soil. This concentrated flow increases the speed of water and erosive power, and thus more effectively transports water, solutes, and sediment downstream (Doyle and Bernhardt 2011). Furthermore, drainage ditches have altered groundwater flow paths by lowering the water table, increasing bulk density of soils, and decreasing hydraulic conductivities (Silins and Rothwell 1998; Price et al. 2003). In fact, the subsidence of the peat layer is likely the most important factor causing ditches to become shallow (Heikurainen 1957). The resulting change in physical properties of catchments after ditching affect the hydrological functioning of these ecosystems (Silins and Rothwell 1998; Price et al. 2003; Holden et al. 2004), including the patterns and strength of hydrological connections between land and water during different runoff conditions (Jencso and McGlynn 2011). The increased canopy cover of trees and shrubs on drained land also changes groundwater conditions by increasing evapotranspiration (Price et al. 2003; Koivusalo et al. 2008), increasing interception (Price et al. 2003) and reducing groundwater recharge.

In addition to these hydrological changes, forest ditches also influence water quality. For example, ditching may result in 1000-fold increases in suspended sediment concentrations and total yield in peat soils with underlying sand (Painter et al. 1974). Bedload measurements suggest that even after ditches mature, erosion on steeper slopes produces substantial changes in the supply of sediment (Painter et al. 1974; Stenberg et al. 2015). The effects of ditching on stream chemistry are also well documented and include greater concentrations of ammonium and organic N (Lepistö et al. 1995; Prevost et al. 1999), heavy metals (Holden et al. 2004; Annala et al. 2014), and micronutrients (Åström et al. 2001), as well as elevated pH (Prevost et al. 1999; Åström et al. 2001), and increased water temperature (Prevost et al. 1999). These changes may be the result, at least in part, of lowering of the water table and aerating previously inundated peat soils, which in turn affects microbial processes and decomposition rates (Holden et al. 2004).

To function as intended, drainage ditches may require periodic maintenance (Päivänen and Hånell 2012), but the potential positive effects of maintenance on tree productivity must be weighed against economic and environmental costs. Ditch network maintenance (DNM), or the cleaning out of existing ditches of vegetation, eroded soils, or other debris, can influence water quality similar to that of new forest ditching (Manninen 1998; Joensuu et al. 2002; Koivusalo et al. 2008; Hynninen et al. 2011; Stenberg et al. 2015). Indeed, DNM can also have negative effects on aquatic communities (Hansen et al. 2013). As there is a tradeoff between the negative environmental effects of the ditching and tree productivity, as well as a financial cost of the work, DNM should be applied conservatively. The most recent review of the literature on DNM notes that there is a lack of a robust, standard method for assessing the need for DNM (Sikström and Hökkä 2016). Knowing where and when it is worth the risk of negative environmental effects from DNM will be an important step towards ensuring we can meet timber production goals in a cost-effective way while balancing other ecological and water quality issues.

There are currently no clear guidelines for DNM that are oriented toward striking the balance between forest growth and environmental quality (Sikström and Hökkä 2016). Assessing the functional role of ditches and making decisions about DNM requires a clear understanding of (1) where ditches are located and (2) ditch hydrology, including the timing and duration of seasonal flow. In Sweden, ditches are poorly mapped and the published numbers are only approximations of the length of all ditches constructed (Päivänen and Hånell 2012, Sikström and Hökkä 2016). One advantage of high-resolution LiDAR Digital Elevation Models (DEMs) that are currently being created for whole countries (e.g. Sweden, Finland, Poland, Slovenia), is that they contain information about small-scale features such as ditches. Additionally, upslope catchment area (CA) algorithms and topographic indices based on DEMs have shown great promise for helping us better identify and manage small streams, riparian buffers, wet areas, and groundwater hotspots during forestry operations (Ågren et al. 2014, 2015; Kuglerová et al. 2014; Laudon et al. 2016).

The purpose of this study was to take the first steps towards better understanding how to decide which ditches to maintain. We asked the following questions: (1) did workers dig ditches in all soil types or did they make ditches in soils that are more likely to benefit from draining (i.e. peat)? and (2) do all ditches have the potential to drain water? Our study builds on previous research from the Krycklan Catchment Study (KCS) in northern Sweden on the timing and duration of seasonal flow, along with research on flow initiation threshold areas (Ågren et al. 2014, 2015), and uses newly produced high-resolution LiDAR (Light Detection and Ranging)-based DEMs. We hypothesised that there are a large number of ditches on soil types that do not benefit from added drainage, likely because workers were paid to dig ditches regardless of their location (the case of ‘thoughtless ditching’). Furthermore, we hypothesised that there are a large number of ditches that, although they may have been dug in soils that could benefit from added drainage, that they do not have sufficient CA to move water regardless of the condition of the ditch (the case of ‘poorly-planned ditching’).

## Materials and methods

### Study area

**Fig. 1:**
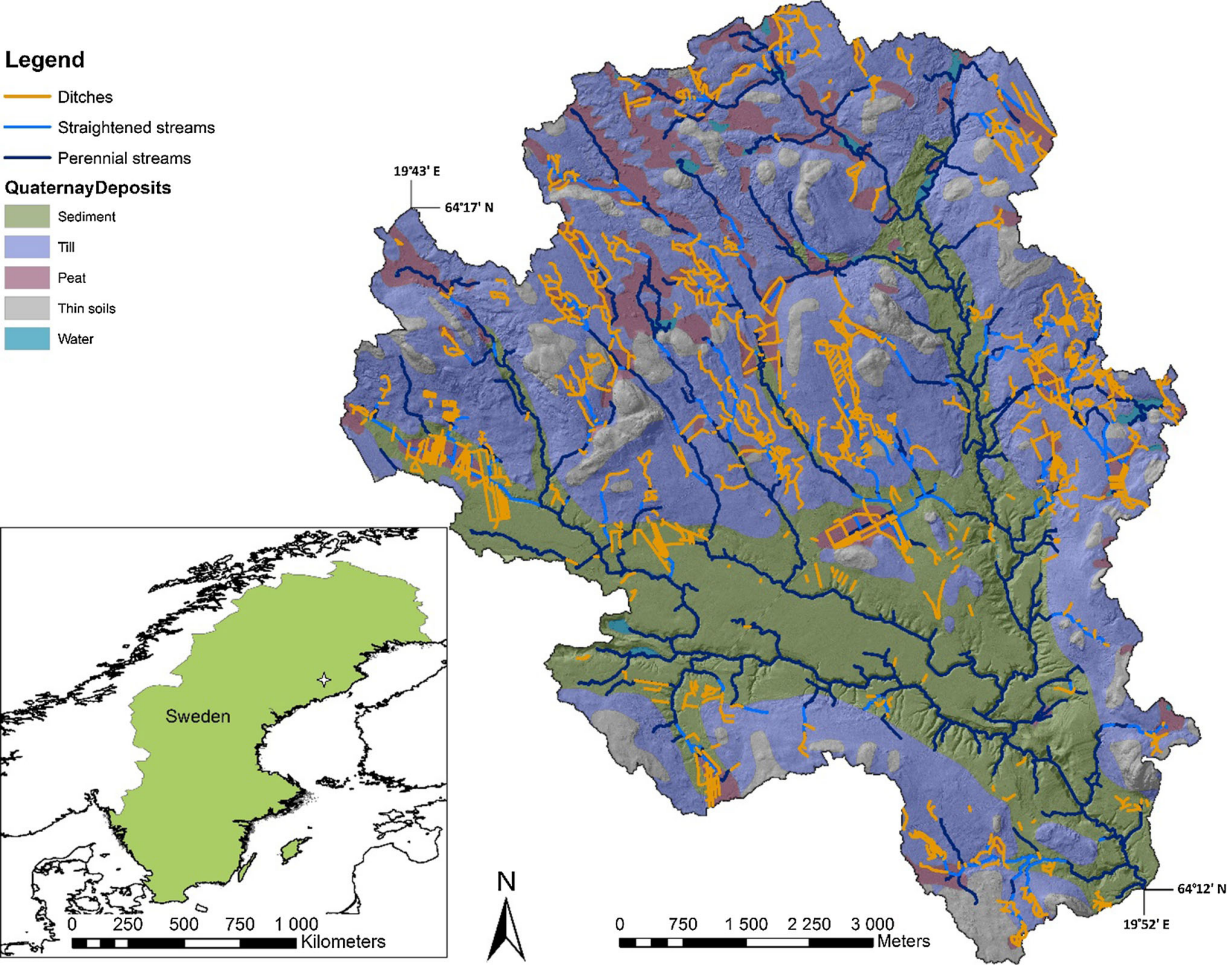
Location map of study area in northern Sweden. The star on the inset map shows the approximate location of the detailed map. The detailed map displays the outline of the KCS with a hillshade rendition of the topographic relief along with drainage ditches, straightened perennial streams, all perennial streams, and soil types. Latitude and longitude are also noted

Krycklan is a tributary to the Vindel River in northern Sweden, approximately 60 km north-west of the city of Umeå (Fig. 1; Laudon et al. 2013). The 68 km^2^ Krycklan Catchment Study (KCS) is relatively typical for the region and has a low relief, varying from 138 to 339 m.a.s.l. (Laudon et al. 2013). The most recent glaciations have resulted in post-glacial isostatic rebound, which has caused land upliftment of approximately 250 m above the current sea level and has divided the landscape by the former highest coastline (FHC), above which glacial legacy till remains unsorted, and below which larger heterogeneity in the parent material exists from washed till to large deposits of sorted glaciofluvial sediments (Fig. 1; Laudon et al. 2013). The northern part of the KCS is above the FHC, and the southern part below the FHC. The KCS is dominated by secondary forests with a dominance of Scots pine (*Pinus sylvestris* L.) and Norway spruce (*Picea abies* L. H. Karst.) with birch (*Betula pubescens* Ehrh.), alder (*Alnus incana* (L.) Moench), aspen (*Populus tremula* L.), and willows (*Salix* spp.) found in mesic-wet and riparian habitats. Land use is dominated by forestry with clear-cuts representing about 7% of the catchment. Arable land and built areas represent only about 2% of the catchment. Although mapped mires only encompass 9% of the KCS, much of the forested area has been ditched and has overlying peat soil, but it is currently < 50.5 cm thick. These layers could have been thicker previously, but have compacted with draining, and hence are mapped as ‘till soil’ in the Swedish system. The mean annual temperature is 1.8 °C and average precipitation is 614 mm of which about 40% falls as snow. The hydrology is snowmelt driven with peak flows occurring during spring flood, usually in May (Laudon et al. 2013). The KCS is an ideal test case for this effort because of the history of hydrological and biogeochemical research, existing field data, and the availability of a high-resolution LiDAR-based DEM.

### Ditch characterisation

A LiDAR point cloud with 3.3–10.2 points/m^2^ was used to create a DEM at 0.5 × 0.5 m resolution for the KCS. Analytical hillshade models from different angles were used to manually digitalize ditch channels as polylines in ArcMap 10.3. Ditches that were unclear from hillshade layers were verified in the field. We categorised ditches into ‘straightened streams’ when they overlapped the modelled perennial stream network (> 10 ha CA; Ågren et al. 2014). We determined the length and percentage of ditches and straightened streams on different soil types within the KCS by grouping soils into four categories based on surveys done by the Geological Survey of Sweden Quaternary Deposits: thin soils and boulder outcrops (Ågren et al. 2014), fluvial sediments, glacial till, and peat (Geological Survey of Sweden Quaternary Deposits map designates peat as > 50.5 cm). We then calculated the length and density of ditches and streams for each soil type.

### Pre-processing of DEMs and basic description of upslope CA algorithms

The national DEM from the Swedish Mapping, Cadastral, and Land Registration Authority was used as the basis for the upslope CA algorithms. This DEM is created from a point cloud with a density of 0.5–1 point/m^2^ and has a cell resolution of 2 × 2 m. All bridges and culverts were manually mapped in the field with a GPS to hydrologically correct the DEM and the mapped ditches were burned into the DEM and given a 1 m depth (Whitebox GAT 3.3). This manually adjusted DEM was then breached using Go-Spatial (Lindsay 2016) in order to solve remaining sinks and create a flow compatible DEM. This new hydrologically corrected DEM was then used as input into flow direction and accumulation models using the Deterministic 8 (D8) algorithm (O’Callaghan and Mark 1984). Because these models are based on DEMs, they assume that subsurface water flowpaths follow surface topography, which is usually the case in forested catchments draining former glaciated landscapes (Rodhe and Seibert 1999; Ågren et al. 2014) and is reasonable for other environments as well (Jencso and McGlynn 2011).

By varying the flow initiation threshold in GIS calculations, the stream network during different flow conditions can be mapped (Ågren et al. 2015). Smaller flow initiation thresholds (e.g. 1 ha) predict conditions at high flow, while larger flow initiation thresholds (e.g. 10 ha) predict conditions at low flow (Ågren et al. 2015). We compared two methods of upslope CA algorithms to determine the probability of ditches to support flowing water, and thereby assess ditch capacity to drain forests: the CA-method and the stream network overlap (SNO)-method.

### Catchment area (CA) method

First, we calculated the CA of each ditch segment using the “add grid values to shapes” tool with the upslope CA as a grid in SAGA GIS (Conrad et al. 2015). To determine which ditches would remain dry at even high flow conditions (i.e. spring flood), we compared the CA’s of our ditch segments with the flow initiation threshold areas tested in the field by Ågren et al. (2015). They found that at high flow conditions, the average CA resulting in flow initiation was 1 ha (range 0.4–4.4 ha) if land was drained by a stream or ditch. Accordingly, if a ditch segment had a CA less than 0.4 ha, it is highly unlikely to support flowing water.

### Stream network overlap method (SNO)

The second method we used to assess the probability of ditches supporting flowing water was to model the spatial extent of the stream network using upslope CA algorithms (Ågren et al. 2014) focusing on the flow initiation threshold areas for high flow (1 ha, with a range of 0.4–4.4 ha). We then evaluated the degree of overlap between the modelled stream network and the actual location of ditches using the “intercept” tool in ArcGIS. Ditches that did not overlap with the stream network at high flow, and thus were not modelled to have running water at this time of year when water is most likely to be flowing, were considered inactive.

## Results

### Ditch characterisation

There are about 150 km of ditches within the 68 km^2^ (6790 ha) KCS (Fig. 1; Table 1). Most ditches were mapped on till soils (57%), and the fewest mapped onto thin soils (1.4%; Table 1). This is in contrast to the density of ditches, where the highest mean densities of ditches were found on deep peat soils (5.88 km/km^2^), again with the least dense ditch configurations on thin soils (0.37 km/km^2^; Table 1). On average, there are 2.21 km of ditches per km^2^ of land (Table 1). Overall, there are 0.84 km of ditches per km of perennial stream (10 ha flow initiation point; Table 1), with the highest ratio being in till soils (1.46) and the lowest on sediment (0.28; Table 1). Furthermore, 20% of the perennial streams have been straightened and were likely ditched at some point in the past.

**Table 1:** Length of ditches and streams in each soil type within the KCS (includes all land cover types). Waterbodies are excluded, thus the catchment area is less than 68 km^2^

Soil type	Area (km^2^)	Km of ditches	Mean (min–max) density of ditches (km/km^2^)	% of ditches	Km of straightened perennial streams^a^	Km of all perennial streams^a^	% perennial streams^a^ straightened	Ditch:stream
Till	34.37	84.27	2.45 (0.01–9.08)	56.59	19.61	57.57	34.06	1.46
Peat	6.34	37.28	5.88 (0.41–28.91)	25.03	9.14	29.06	31.45	1.28
Sediment	20.85	25.22	1.21 (0.10–4.42)	16.94	9.5	90.19	10.53	0.28
Thin soils	5.81	2.15	0.37 (0.08–8.79)	1.44	0.47	1.27	37.01	1.69
All	67.37	148.92	2.21 (0.01–28.91)	100	38.72	178.08	21.74	0.84

^a^Perennial straightened streams are cleaned/ditched channels with greater than a 10-ha catchment area (Ågren et al. 2015)

### Upslope CA algorithms

**Fig. 2:**
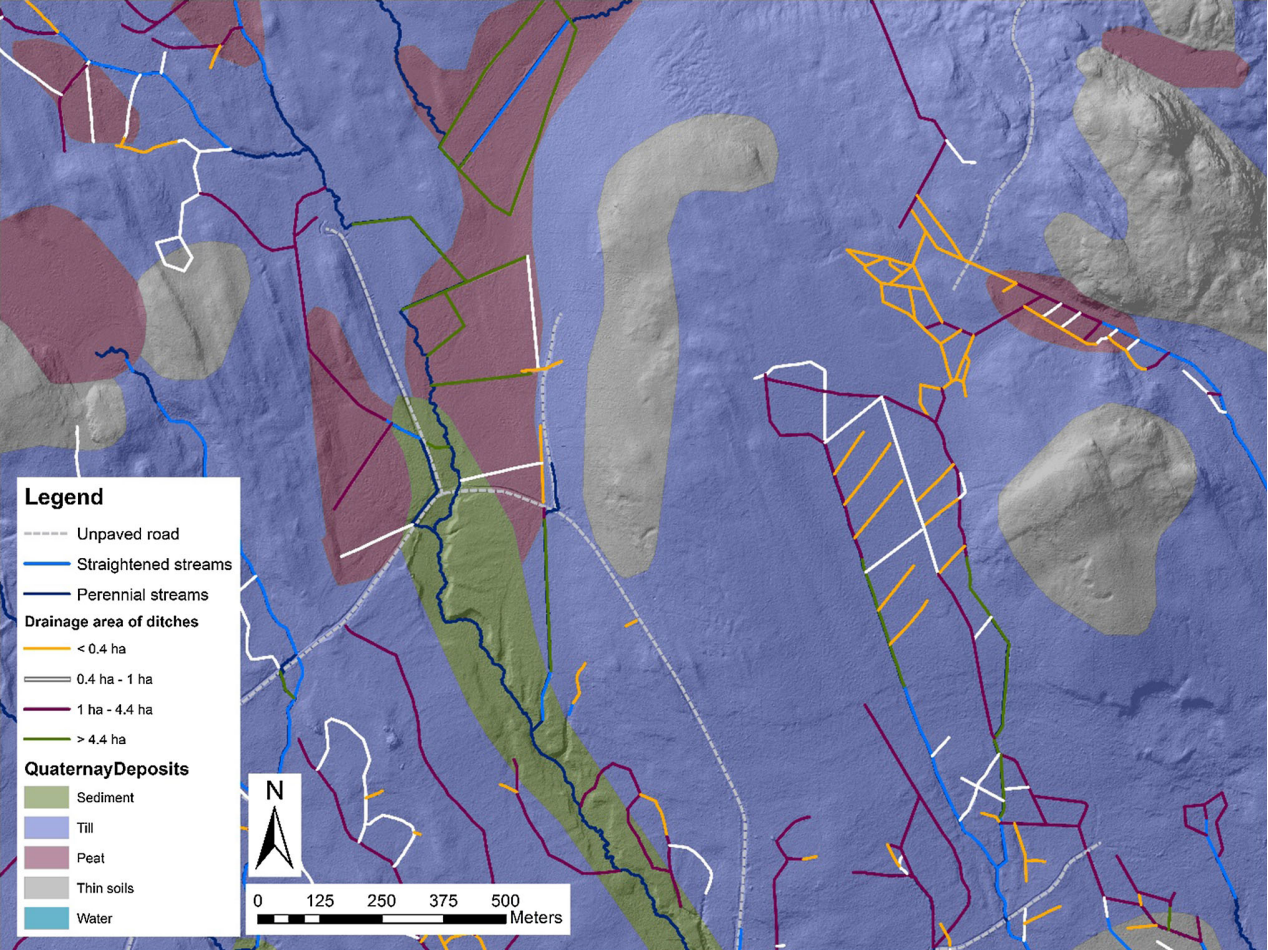
Drainage ditches and 10 ha streams overlaid on soil type. Ditches are colour coded by their catchment areas (CA): 0.4 ha is the smallest, 1 ha is the mean, and 4.4 ha is the largest flow initiation size during a high flow event based on Ågren et al. (2015). 10 ha streams that have been straightened are also mapped

**Fig. 3:**
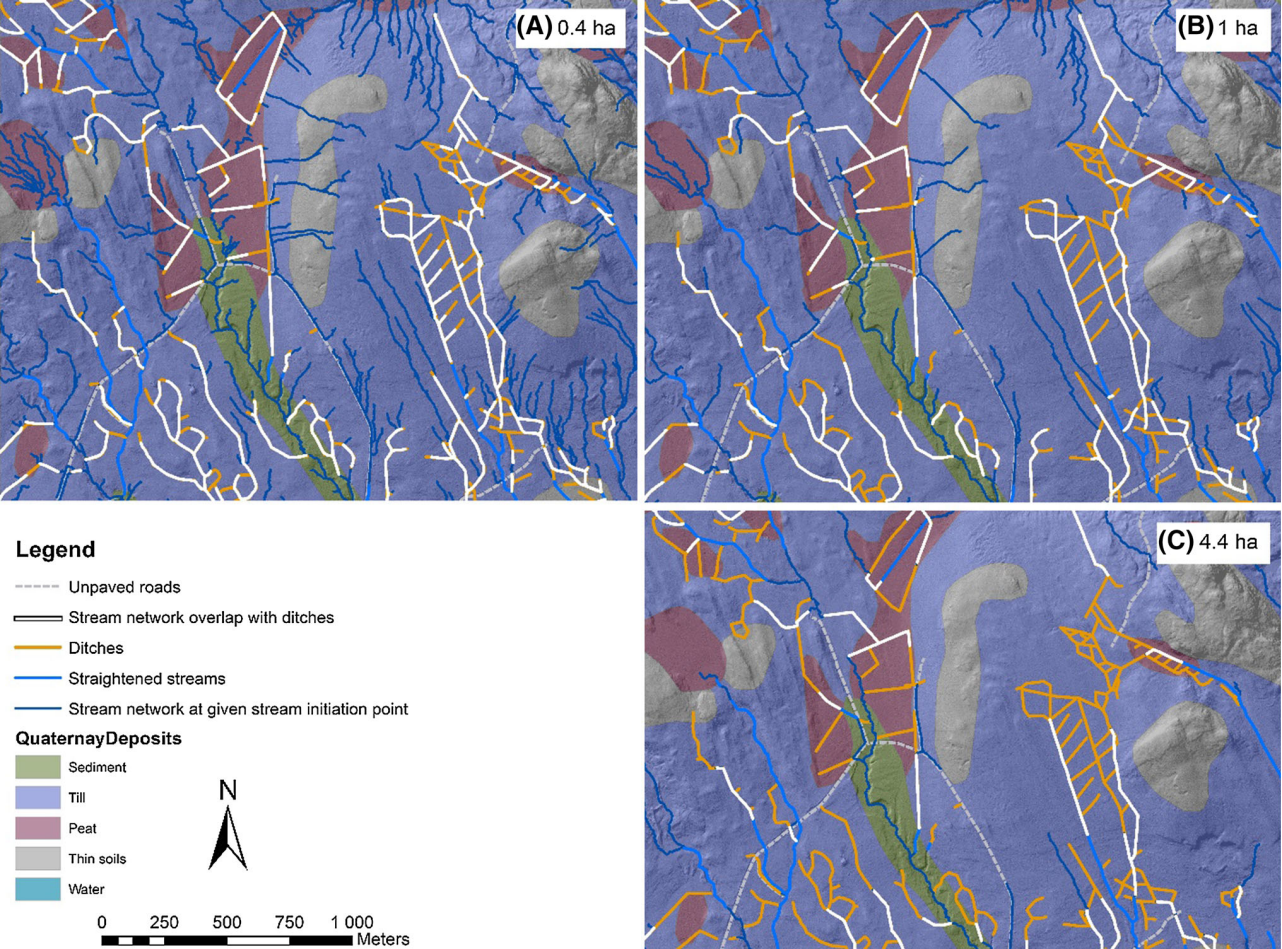
Map of the stream network overlap (SNO) of forest ditches and **a** the 0.4 ha stream network, the smallest flow initiation point for high flow events; **b** the 1 ha stream network, the mean flow initiation point for high flow events; and **c** the 4.4 ha stream network, the maximum flow initiation point for high flow events. If the stream network did not flow in ditches, it was assumed to be dry

When using the most conservative CA to initiate flowing water at the KCS (0.4 ha, Ågren et al. 2015), our model showed that 25–29% of all ditches were inactive even during peak flow events, including the spring flood when flows are on average 225% higher than summer base flows (Karlsen et al. 2016; Table 2; Figs. 2, 3). By comparison, using the average CA needed to initiate flowing water for ditches at the KCS (1 ha, Ågren et al. 2015), 46–51% of ditches were modelled to be inactive during peak flow events (all ditches with CA sizes of < 0.4 ha combined with 0.4–1 ha, Table 2). At the high end of the CA needed to initiate flowing water (4.4 ha, Ågren et al. 2015), 75–92% of ditches were modelled to be inactive (all ditches with CA sizes < 4.4 ha combined, Table 2). When comparing the two methods, the CA-method and the SNO-method yielded more similar results at the lower flow initiation threshold areas than at the larger (Table 2). The CA-method predicted 16% fewer inactive ditches than the SNO-method at the most conservative flow initiation threshold of 0.4 ha, 50% more inactive ditches at the lower range (0.4–1 ha) flow initiation thresholds, 41% more inactive ditches when modelling the upper range (1–4.4 ha), and 69% fewer inactive ditches when maximum flow initiation thresholds were modelled (4.4–10 ha; Table 2).

**Table 2:** Comparison of the length and total percentage of ditches that are inactive (no flowing water) at high flow within the KCS based on two methods: (1) catchment area (CA) method or (2) stream network overlap (SNO) method, i.e. using a given CA for flow initiation threshold area and determining the degree of overlap with the ditch network. The absolute minimum flow initiation threshold area found in Ågren et al. (2015) during high flow events was 0.4 ha, the mean for locations with drainage ditches was 1 ha, the maximum was 4.4 ha, and perennial streams have CAs of 10 ha

Method	Flow initiation area (ha)	Inactive (km)	Inactive (%)	Cumulative % inactive
CA	< 0.4	37	25	25
SNO	< 0.4	44	29	29
CA	0.4–1	39	26	51
SNO	0.4–1	26	17	46
CA	1–4.4	62	41	92
SNO	1–4.4	44	29	75
CA	4.4–10	11	7	100
SNO	4.4–10	36	24	100

## Discussion

We found that there are almost as many ditches as there are perennial streams within the KCS. Furthermore, many perennial streams within the KCS were also identified as straightened, likely to increase their drainage capacity. Indeed, it is noteworthy that in these remote forest landscapes—which at first glance appear far less altered than urban and agricultural counterparts—have drainage systems that have been highly modified. It has already been shown that 76% of the actual stream network is missing on the most current detailed Swedish topographic map (1:12,500; Ågren et al. 2015) and this is a widespread problem often limited by traditional mapping methods that use aerial photography and satellite imagery (Benstead and Leigh 2012). The addition of ditches adds another large component of small stream-like features to the drainage structure of Sweden and likely changes the location of many of the modelled streams. It is difficult to know how many of these ditches were naturally small ephemeral streams activated only occasionally before ditching commenced; regardless, these ditches are now extensions of the stream network and have almost doubled its size.

After this extensive ditching, the stream network geometry is also very different than it was naturally, with dendritic stream network structures transformed to lattices, grids or comb-like systems in the headwaters (Fisher et al. 2004; Figs. 1, 2, 3). Such widespread changes to channel geometry have potentially had dramatic ecological and biogeochemical consequences in these landscapes that merit attention from researchers. For example, the movement of aquatic and riparian organisms may be restricted to corridors (i.e. watercourses) of suitable habitat (Lõhmus et al. 2015). How close these corridors are to each other can influence how frequently overland dispersal occurs and thus shape metacommunity structure in river networks (Brown and Swan 2010; Göthe et al. 2013; Kuglerová et al. 2015), if indeed these ditches provide suitable habitat (Lõhmus et al. 2015). Furthermore, stream network geometry determines the distribution of distances water and solutes travel from points of input on land to the nearest channel (i.e. along terrestrial flowpaths) as well as the time spent in small channels prior to exiting catchments. In this way, major reconfiguration of network structure can affect the overall catchment residence time of solutes and the potential for instream or hyporheic processing within the drainage system (Mallard et al. 2014). Additionally, the number and density of tributary confluences could influence biogeochemical dynamics such as nutrient retention efficiency (Fisher et al. 2004) and sediment distribution and transport (Benda et al. 2004). In a temporal perspective, the presence of a ditch network typically makes hydrographs flashier with higher peak flows and less temporary storage in response to high flow events (Holden et al. 2004) likely resulting in a shorter time window of connectivity between land and water. Hence, ditches have fundamentally altered the hydrological connectivity with major implications for the hydrology, water chemistry, and plant and animal communities that live in and around these watercourses.

In the context of sustainable forest management, disturbance to ditches must be evaluated before considering DNM because this disturbance will be disproportionately transferred downstream (Doyle and Bernhardt 2011). Furthermore, the effects to surrounding the forest due to the change in hydrology must also be assessed for biodiversity because ditching primarily targets living organisms (notably trees and soil biota) through their limiting factors, i.e. water availability (Lõhmus et al. 2015).

In many countries, decision making for ditching or DNM is almost solely based on site type and estimated timber productivity (considering geographical location; Sikström and Hökkä 2016), but rarely considers hydrological parameters. Although a growing body of literature from Finland is trying to account for the effect of evapotranspiration on drained peatland hydrology (Koivusalo et al. 2006; Sarkkola et al. 2013), they typically do not consider the CAs of specific ditches in their models (e.g. FEMMA; Laurén et al. 2005; Koivusalo et al. 2006). For example, Sarkkola et al. (2013) suggested that no DNM would be necessary in mature stands in central and southern Finland, even if the condition of the ditch network was poor because growing-season precipitation is transferred back to the atmosphere by forest evapotranspiration. While in northern Finland, DNM was considered important in low-stocked sites (< 100 m^3^ ha^−1^) to control drainage conditions because of the low evapotranspiration potential (Sarkkola et al. 2013). Studies that have included CAs are typically those dedicated to evaluating the erosion potential of ditch networks. For example, Holden et al. (2007) showed that channel slope and the upstream CA explained most of the observed variation in erosion occurring across ditched sites. Further, consideration of CA in combination with evapotranspiration modelling using the FEMMA model (Koivusalo et al. 2006) has been successfully used to assess erosion from ditch networks (Haahti et al. 2014) as well as the air-filled porosity of the rooting zone in peatland soils (Haahti et al. 2016). Thus, there is promising research into forest hydrology that may shed light on how we can manage forest stand volumes in order to avoid DNM, but this has not yet been applied at the scale of specific ditches.

### Ditches by soil type, evaluating ‘thoughtless ditching’

The density of ditches differed by soil type, presumably because workers targeted soils and areas that were in need of most drainage. However, as hypothesised, there were a number of ditches on soil types that likely do not benefit from added drainage. This ‘thoughtless ditching’, was located in sedimentary or thin soils that generally have high hydraulic conductivities and drain quickly and deeply (Koch et al. 2011), or are so thin that ditches will likely not help increase tree growth. It may be that workers were paid to dig ditches regardless of their location, and thus, one approach to prioritising DNM would be to map the ditches and compare them to soil types. Doing this, we can recommend that 17% of ditches be actively or passively restored back to their pre-ditch state (sedimentary + thin soils; Table 1). Furthermore, erosion may more severe in the ditches cut into mineral subsoil than in ditches cut into thick peat (Stenberg et al. 2015); thus, not only should DNM be avoided in these soils due to their quick draining properties, but also because they erode more easily.

Ditches in till soils almost all have overlying peat but are classified as the underlying mineral soil type because the Swedish Geological Society soil map that we used defines peat as > 50.5 cm (Table 1), therefore even more of these areas would be classified as peatlands in other countries. Areas with < 30 cm of peat would be called shallow peatlands in Finland, which make up 20.4% of the all peatlands in Finland, of which 58.3% have been drained for forestry (Korhonen et al. 2013). Thus, research in Finland is relevant for our study system, with some caution. On these shallow peatlands, initial drainage may be more or less permanent, preventing the re-paludification process (Päivänen and Hånell 2012). Thus, there is likely less potential for DNM needed in these shallow peatlands, but upslope CA algorithms are crucial to be more certain of their potential to drain forest water.

### Modelling ditch inactivity, evaluating ‘poorly-planned ditches’

We hypothesised a priori that there are a large number of ditches that, although they may have been dug in soils that could benefit from added drainage (i.e. peat or shallow peat), they do not have sufficient CA to move water regardless of the condition of the ditch (the case of ‘poorly-planned ditching’). We evaluated two methods that use upslope CA algorithms, the CA-method and the SNO-method for determining if ditches are inactive across the whole KCS, regardless of soil type. The CA-method and the SNO-method yielded more similar results at the lower flow initiation threshold areas than at the larger, likely because the CA-method is an average of the CA over the length of the ditch segment and thus has error built into the measurement. The intervals of the flow initiation areas we report are increasingly wider, and thus, the error from taking an average in the CA-method increases. The SNO-method is more accurate in that it predicts precisely where flowing water would start within the ditch network. With this in mind, we supported our hypothesis, and at least 25% of ditches in the KCS likely do not carry flowing water, even during peak flow events (< 0.4 ha flow initiation CA), and thus serve no contemporary purpose. Given that 0.4 ha is the minimum CA at which a ditch would be active (Ågren et al. 2015) and that at this threshold the variation among methods was low; any ditches that have < 0.4 ha CA size would be a conservative threshold for ditch inactivity and thus, a strong candidate for *not* being maintained. Ditches with between 0.4–1 ha CAs would also be good choices when avoiding DNM because most flow initiation points during high flow events from Ågren et al. (2015) were larger than 1 ha, even when ditches were considered. Thus, between 46 and 51%, when including both the < 0.4 ha and 0.4–1 ha CA flow initiation ranges, of ditches within the KCS could be left unmaintained and/or might be candidates for ecological restoration.

One critique of this approach could be that the ditches with small CAs may only be active and important after forest harvest when evapotranspiration is lower, thus raising the water table and hampering tree growth (Sikström and Hökkä 2016). Ågren et al. (2015) tried to model the expansion of groundwater discharge areas after clear-cutting using K-values (saturated hydraulic conductivity of the soil layer in m s^−1^), but found their use to be difficult to measure and inaccurate. Using experience from recent studies from a nearby catchment that underwent a clear-cut harvest, it is likely that flow would, at a maximum, increase by about 30% during high flow events (Sørensen et al. 2009; Schelker et al. 2013). However, there is high inter-annual variation in this response, and clear-cutting could have no effect on runoff during some conditions (Schelker et al. 2013). On average, a 1 ha CA was needed to initiate a water flow in ditches during high flow events in Ågren et al. (2015), therefore using the very conservative 0.4 ha that is 60% lower threshold for flow initiation, even in clear-cut conditions ditches should be inactive. But these assumptions require field testing under different forest ages and across the different soil types to be certain.

### Importance and ease of ditch mapping and upslope CA algorithms

Although the models we used to determine the activity of ditches were developed in the 1970s and 80s (O’Callaghan and Mark 1984), the widespread availability of high-resolution DEMs and free GIS software makes their application less expensive, easier to use and more accurate than what was previously possible. In the past, DEMs were only available at 50 × 50 m scale, making upslope CA algorithms too coarse. Now, many countries have nationwide efforts to generate high-resolution LiDAR images. There are currently efforts by the Swedish Forest Agency to map ditches using LiDAR images manually as we did, as well as using image recognition software used previously in agrarian landscapes (Bailly et al. 2008). Their efforts are producing information about the depth and slope of the ditches that could even better refine our models. With these high quality DEMs, combined with free GIS software, one can easily create the high-resolution stream network models and ditch upslope CA algorithms. Thus, this mapping can be done by forest companies, management agencies or landowners, making future management of these small watercourses much more informed.

### Soil type (thoughtless ditching) versus upslope CA algorithms (poorly-planned ditching)

We have discussed two approaches to deciding which ditches should undergo DNM based on: soil type (thoughtless ditching; Table 1) or upslope CA algorithms (poorly-planned ditching; Table 2). There are assumptions and error included in both approaches and forest managers will need to consider which approach works best for them based on data availability and accuracy. For example, soil maps are often readily available for download, but they are usually delineated from aerial photographs and could have substantial error built into them. For upslope CA algorithms, the availability and cost of high-resolution DEMs could prevent accurate mapping of ditches and subsequent modelling. One improvement of the CA-method would be to split the ditches into smaller segments based on the scale at which managers are deciding DNM activities (e.g. 10, 50 or 100 m lengths) in order to be more accurate as well as make the method more practical for on-the-ground workers.

## Conclusions

By identifying ditches from new LiDAR-derived DEMs and applying upslope CA algorithms to model their potential to carry flowing water, we have used a novel approach to try to reconcile timber production, water quality and ecosystem conservation (sensu Lõhmus et al. 2015). Before this, there were no published guidelines for DNM oriented toward striking a balance between forest growth and environmental quality. We have taken a first step towards identifying forest drainage ditches that are candidates for *not* being maintained. First, we located ditches using LiDAR-derived DEMs and found that ditches nearly doubled the size of the stream network (from 178 km to 327 km) within the KCS, a landscape that many would describe as relatively pristine. National LiDAR datasets are being generated throughout Europe and our methods could have widespread application for identifying previously unknown forest ditches. Next, by identifying ‘thoughtless ditching’, likely done because private forest owners were paid to dig ditches as part of government work programmes, up to 17% of ditches could be left unmaintained due to being located in well-draining soil types. Finally, using upslope CA algorithms to identify ‘poorly-planned ditches’, between 25 and 51% of ditches could be left unmaintained because they do not have enough CA to initiate flow. Future work should focus on field testing these methods to explore how flow initiation thresholds relevant for the KCS apply to areas with different climates, forest ages and soil types as well as incorporate costs (i.e. DNM work and interest rates) and final-cutting criteria (energy wood vs. saw logs) into long-term simulations.
